# DNMT3b overexpression contributes to a hypermethylator phenotype in human breast cancer cell lines

**DOI:** 10.1186/1476-4598-7-15

**Published:** 2008-01-25

**Authors:** J Devon Roll, Ashley G Rivenbark, Wendell D Jones, William B Coleman

**Affiliations:** 1 Department of Pathology and Laboratory Medicine, University of North Carolina, Chapel Hill, NC 27599, USA; 2 Department of Biochemistry and Biophysics, University of North Carolina, Chapel Hill, NC 27599, USA; 3 UNC Lineberger Comprehensive Cancer Center, University of North Carolina School of Medicine, Chapel Hill, NC 27599, USA; 4 Expression Analysis, 2605 Meridian Parkway, Durham, NC 27713, USA

## Abstract

**Background:**

DNA hypermethylation events and other epimutations occur in many neoplasms, producing gene expression changes that contribute to neoplastic transformation, tumorigenesis, and tumor behavior. Some human cancers exhibit a hypermethylator phenotype, characterized by concurrent DNA methylation-dependent silencing of multiple genes. To determine if a hypermethylation defect occurs in breast cancer, the expression profile and promoter methylation status of methylation-sensitive genes were evaluated among breast cancer cell lines.

**Results:**

The relationship between gene expression (assessed by RT-PCR and quantitative real-time PCR), promoter methylation (assessed by methylation-specific PCR, bisulfite sequencing, and 5-aza-2'deoxycytidine treatment), and the DNA methyltransferase machinery (total DNMT activity and expression of DNMT1, DNMT3a, and DNMT3b proteins) were examined in 12 breast cancer cell lines. Unsupervised cluster analysis of the expression of 64 methylation-sensitive genes revealed two groups of cell lines that possess distinct methylation signatures: (i) hypermethylator cell lines, and (ii) low-frequency methylator cell lines. The hypermethylator cell lines are characterized by high rates of concurrent methylation of six genes (*CDH1, CEACAM6, CST6, ESR1, LCN2, SCNN1A*), whereas the low-frequency methylator cell lines do not methylate these genes. Hypermethylator cell lines coordinately overexpress total DNMT activity and DNMT3b protein levels compared to normal breast epithelial cells. In contrast, most low-frequency methylator cell lines possess DNMT activity and protein levels that are indistinguishable from normal. Microarray data mining identified a strong cluster of primary breast tumors that express the hypermethylation signature defined by *CDH1*, *CEACAM6, CST6, ESR1, LCN2*, and *SCNN1A*. This subset of breast cancers represents 18/88 (20%) tumors in the dataset analyzed, and 100% of these tumors were classified as basal-like, suggesting that the hypermethylator defect cosegregates with poor prognosis breast cancers.

**Conclusion:**

These observations combine to strongly suggest that: (a) a subset of breast cancer cell lines express a hypermethylator phenotype, (b) the hypermethylation defect in these breast cancer cell lines is related to aberrant overexpression of DNMT activity, (c) overexpression of DNMT3b protein significantly contributes to the elevated DNMT activity observed in tumor cells expressing this phenotype, and (d) the six-gene hypermethylator signature characterized in breast cancer cell lines defines a distinct cluster of primary basal-like breast cancers.

## Background

Inappropriate gene silencing resulting from aberrant DNA methylation significantly contributes to neoplastic transformation, tumorigenesis, and tumor progression [1,2], contributing to some of the hallmarks of cancer [3]. While abnormal DNA methylation affecting a variety of genes occurs in nearly every type of cancer that has been evaluated, some tumors exhibit aberrant concurrent hypermethylation of numerous genes, a phenomenon known as the CpG island methylator phenotype (CIMP). CIMP was first described in a distinct subset of human colorectal carcinomas that displayed high rates of concordant methylation of specific genes [4]. Subsequently, CIMP has been described in other human neoplasms, including tumors of the ovary [5], bladder [6], prostate [6], stomach [7], liver [8], pancreas [9], esophagus [10], and kidney [11], as well as neuroblastomas [12], and leukemias and lymphomas [13,14]. While tissue type is important in determining which genes are targeted for methylation in a given neoplasm, CIMP-positive tumors in each of these tissue types exhibit gene silencing that is due to cancer-specific (rather than age-specific) hypermethylation of epigenetically-regulated genes. Definitive evidence for a hypermethylation defect (similar to CIMP) among human breast cancers has not emerged, and some investigators have suggested that such a hypermethylator phenotype does not occur in breast tumors [15]. Nevertheless numerous epigenetically-regulated genes are known to be directly silenced by DNA methylation in breast cancer including cell cycle control genes (*APC, RASSF1, RB, TFAP2A*), steroid receptor genes (*ESR1, PGR, RARα*), tumor suppressor genes (*BRCA1, CDKN2A, CST6*), and metastasis-associated genes (*CDH1, CEACAM6, PCDHGB6*), among others [16-19].

In the current study, we analyzed 12 breast cancer cell lines for differential expression of 64 methylation-sensitive genes, to determine if subsets of breast cancer cell lines methylate genes at disparate frequencies, and subsequently confirmed that lack of gene expression was attributable to methylation-dependent silencing. Unsupervised cluster analysis of gene expression patterns reveals two distinct groups of breast cancer cell lines that possess different methylation signatures: (i) hypermethylator cell lines, and (ii) low-frequency methylator cell lines. The hypermethylator cell lines are characterized by high rates of concurrent methylation of six genes (*CDH1, CEACAM6, CST6, ESR1, LCN2*, and *SCNN1A*), whereas the low-frequency methylator cell lines typically lack methylation of these genes. Analysis of the enzymes responsible for human DNA methylation reveals aberrant DNMT3b protein expression and elevated total DNA methyltransferase activity in hypermethylator cell lines. These observations combine to suggest the existence of a distinct subset of human breast cancer cell lines that possess novel biological properties related to dysregulation of the methylation machinery resulting in the acquisition of a hypermethylator phenotype.

## Results

### Analysis of Epigenetically-regulated Genes Reveals Two Distinct Expression

#### Patterns Among Breast Cancer Cell Lines

**Figure 1:**
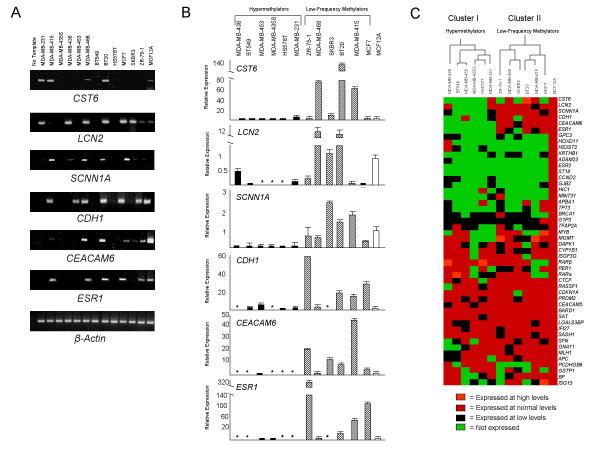
**Expression analysis of methylation-sensitive genes in human breast cancer cell lines**. (A) Representative agarose gels of RT-PCR products from *CST6, LCN2, SCNN1A, CDH1, CEACAM6, ESR1*, and *β-actin*. The source of cDNA template is identified for each lane. Normal breast epithelial MCF12A cells represent the positive control cell line. (B) Quantitative real-time PCR results for *CST6, LCN2, SCNN1A, CDH1, CEACAM6*, and *ESR1*. Black bars correspond to hypermethylator cell lines, cross-hatched bars correspond to low-frequency methylator cell lines, and the white bar (far right) corresponds to MCF12A cells (index control cell line). The expression level of each gene is depicted relative to that of MCF12A cells. Error bars represent S.E.M. Instances of no detectable level of quantitative real-time PCR expression are indicated by an asterisk (*). (C) Unsupervised cluster analysis for 48 genes that are expressed at a detectable level in MCF12A cells. The 12 breast cancer cell lines group into two distinct clusters, designated cluster I (corresponding to hypermethylator cell lines) and cluster II (corresponding to low-frequency methylator cell lines).

Semi-quantitative RT-PCR was performed on a panel of 64 methylation-sensitive genes in each of 12 breast cancer cell lines (BT20, BT549, Hs578T, MCF7, MDA-MB-231, MDA-MB-415, MDA-MB-435S, MDA-MB-436, MDA-MB-453, MDA-MB-468, SKBR3, and ZR-75-1), as well as the normal breast epithelial cell line MCF12A (Figure 1A). Epigenetically-regulated genes that are predictive of CIMP in other tumor systems, as well as genes known to be aberrantly methylated in breast cancer, were selected for expression analysis (Table 1). Levels of expression for each gene in each breast cancer cell line were scored relative to the levels of expression in MCF12A cells: undetected (no expression), low (detectable, but <MCF12A), normal (equivalent to MCF12A), or high (>MCF12A). Quantitative real-time PCR was performed on a subset of epigenetically-regulated genes (n = 6) to confirm the RT-PCR expression results (Figure 1B). This analysis revealed a statistically significant correlation (R = 0.76, p < 0.0001) between the quantitative real-time PCR and RT-PCR results. Gene expression results from the 12 breast cancer cell lines as well as those of MCF12A cells were subjected to an unsupervised cluster analysis, which revealed two distinct groups of six cell lines that differ in their expression of methylation-sensitive genes: cluster I is composed of cell lines (MDA-MB-436, BT549, MDA-MB-453, MDA-MB-435S, Hs578T, and MDA-MB-231) that express a putative hypermethylator phenotype, and cluster II consists of cell lines (ZR-75-1, MDA-MB-468, SKBR3, BT20, MDA-MB-415, and MCF7) that express a putative low-frequency methylator phenotype (Figure 1C). The separation of these two groups is driven predominately by the differential expression of six methylation-sensitive genes (*CDH1, CEACAM6, CST6, ESR1, LCN2*, and *SCNN1A*), which are largely unexpressed by the cell lines in cluster I (putative hypermethylator group), and typically expressed by the cell lines in cluster II (putative low-frequency methylator group).

**Table 1:** Epigenetically Regulated Genes Chosen for Expression Analysis

**Gene Designation**	**Unigene Number**	**Cancer-specific methylation^1^**	**Gene Designation**	**Unigene Number**	**Cancer-specific methylation^1^**
*ADAM23*	Hs.591643	CIMP	*ISG15*	Hs.458485	Breast
*APBA1*	Hs.592974	Breast, CIMP	*ISGF3G*	Hs.1706	Breast
*APBA2*	Hs.525718	Breast, CIMP	*KRTHB1*	Hs.584773	Breast
*APC*	Hs.158932	Breast, CIMP	*LCN2*	Hs.204238	Breast
*BARD1*	Hs.591642	Breast	*LGALS3BP*	Hs.514535	Breast
*BF*	Hs.69771	Breast	*MGMT*	Hs.501522	Breast, CIMP
*BRCA1*	Hs.194143	Breast, CIMP	*MINT31*	AF135531^2^	Breast, CIMP
*C8orf4*	Hs.591849	Breast	*MLH1*	Hs.195364	Breast, CIMP
*CCND2*	Hs.376071	Breast, CIMP	*MYB*	Hs.531941	CIMP
*CDH1*	Hs.461086	Breast, CIMP	*PARP12*	Hs.12646	Breast
*CDKN1A*	Hs.370771	CIMP	*PCDHGB6*	Hs.368160	Breast
*CDKN2A*	Hs.512599	Breast, CIMP	*PER1*	Hs.445534	Breast, CIMP
*CDKN2B*	Hs.72901	CIMP	*PGR*	Hs.368072	Breast, CIMP
*CEACAM5*	Hs.220529	Breast	*PRDM2*	Hs.371823	Breast, CMP
*CEACAM6*	Hs.466814	Breast	*PRKCDBP*	Hs.434044	Breast, CIMP
*CST6*	Hs.139389	Breast	*RARα*	Hs.137731	Breast, CIMP
*CTCF*	Hs.368367	Breast, CIMP	*RARβ*	Hs.536687	Breast, CIMP
*CYP1B1*	Hs.154654	Breast	*RASSF1*	Hs.476270	Breast, CIMP
*DAPK1*	Hs.380277	Breast, CIMP	*RB1*	Hs.408528	Breast, CIMP
*ESR1*	Hs.208124	Breast, CIMP	*SASH1*	Hs.193133	Breast
*ESR2*	Hs.443150	Breast, CIMP	*SSAT*	Hs.28491	Breast
*G1P3*	Hs.523847	Breast	*SCNN1A*	Hs.591047	Breast
*GADD45A*	Hs.80409	Breast	*SERPINB5*	Hs.55279	Breast, CIMP
*GJB2*	Hs.591234	Breast, CIMP	*SFN*	Hs.523718	Breast, CIMP
*GNA11*	Hs.73797	Breast	*SIM*	Hs.520293	Breast
*GPC3*	Hs.567276	Breast	*ST18*	Hs.147170	Breast
*GSTP1*	Hs.523836	Breast, CIMP	*STYK11*	Hs.515005	CIMP
*HIC1*	Hs.72956	Breast, CIMP	*TFAP2A*	Hs.519880	Breast
*HOXD11*	Hs.421136	Breast, CIMP	*THBS1*	Hs.164226	CIMP
*HS3ST2*	Hs.622536	Breast, CIMP	*TMEM45A*	Hs.126598	Breast
*IFI27*	Hs.532634	Breast	*TP73*	Hs.192132	CIMP
*IGFBP5*	Hs.369982	Breast	*WT1*	Hs.591980	Breast, CIMP

^1^"Breast" signifies gene is reported in the literature to be methylated specifically in breast cancer; "CIMP" signifies gene is reported to be methylated among CIMP-positive cancers other than breast, including: colorectal, gastric, hematopoietic, hepatocellular, neuroblastomas, ovarian, pancreatic, and renal malignancies.

^2 ^MINT31 lacks a Unigene number; instead its accession number is provided.

### Methylation Analysis Confirms Epigenetic-regulation of Silenced Genes

**Figure 2:**
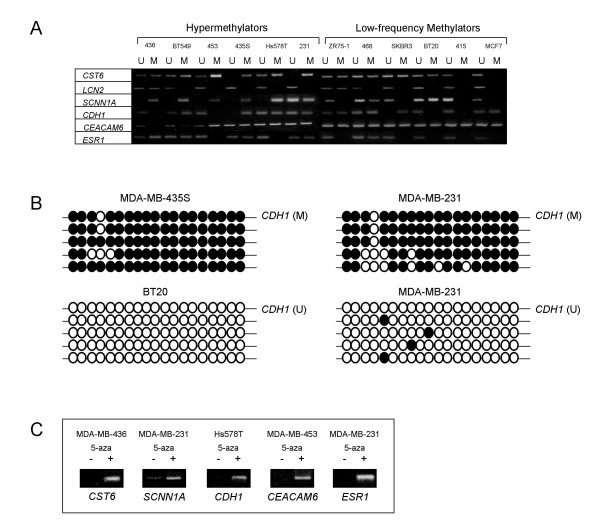
**Methylation analysis of *CST6, LCN2, SCNN1A, CDH1, CEACAM6*, and *ESR1 *among putative hypermethylator and low-frequency methylator cell lines**. (A) Representative agarose gels of methylation-specific PCR (MSP) products corresponding to *CST6, LCN2, SCNN1A, CDH1, CEACAM6*, and *ESR1 *are shown. U = unmethylated MSP product, M = methylated MSP product. Cell line abbreviations are as follows: 231 = MDA-MB-231, 415 = MDA-MB-415, 435S = MDA-MB-435S, 436 = MDA-MB-436, 453 = MDA-MB-453, and 468 = MDA-MB-468. All other cell lines are designated by their full name. (B) Representative bisulfite sequence analysis for *CDH1*. Methylated CpGs are designated by closed circles, unmethylated CpGs are designated by open circles for MDA-MB-435S, BT20, and MDA-MB-231 cell lines (5 replicates each). (C) Representative agarose gels of RT-PCR products for *CST6, SCNN1A, CDH1, CEACAM6*, and *ESR1 *demonstrating 5-aza induction of gene expression in hypermethylator cell lines. RT-PCR results using cDNA template from untreated (-) and 5-aza treated (+) are shown.

To confirm that lack of gene expression of known methylation-sensitive genes among this panel of breast cancer cell lines reflects true methylation-dependent epigenetic silencing, a number of methods were employed to assess gene promoter methylation: (i) methylation-specific PCR (MSP), (ii) bisulfite sequencing, and (iii) response to 5-aza-2'-deoxycytidine (5-aza) treatment. MSP analysis of the six genes (*CDH1, CEACAM6, CST6, ESR1, LCN2*, and *SCNN1A*) that are differentially expressed between hypermethylator and low-frequency methylator cell lines revealed differences in the methylation status of specific CpGs within regulatory regions of each gene's promoter, in accordance with a given cell line's methylator status (Figure 2A). The relationship between gene promoter methylation (as assessed by MSP) and loss of gene expression is strong across all hypermethylator cell lines for the genes examined. For example, the hypermethylator cell lines express *SCNN1A *at undetectable or diminished levels (Figure 1C), and MSP analysis of this gene revealed that 5/6 (83%) of these cell lines produce only a methylated MSP product, while MSP analysis of *SCNN1A *in MDA-MB-231 cells produced unmethylated and methylated products. Conversely, all of the low-frequency methylator cell lines (of which 5/6, 83% express *SCNN1A *at normal levels) produced an unmethylated *SCNN1A *MSP product, and only two of these cell lines (BT20 and MDA-MB-468) produced a detectable methylated MSP product (Figure 2A). Methylated MSP products were detected for at least 50% (3/6) of the genes examined in each of the hypermethylator cell lines. MDA-MB-436 cells produced methylated MSP products for three of the genes analyzed, while BT549 and MDA-MB-435S cell lines displayed methylated MSP products for each of the six genes evaluated (Figure 2A). In contrast, unmethylated MSP products were detected for at least 83% (5/6) of the genes examined in each of the low-frequency methylator cell lines, with MDA-MB-415 cells exhibiting unmethylated products for each of the genes examined.

Selected MSP products were sequenced to examine the methylation status of a greater number of CpGs within regulatory regions of selected genes of interest and to evaluate promoter methylation for genes that produced both unmethylated and methylated MSP products (Figure 2A). The results of the bisulfite sequencing analysis support a direct association between gene promoter methylation and gene expression status in the present panel of methylation-sensitive genes (Figure 2B). For example, hypermethylator cell line MDA-MB-435S lacks detectable expression of *CDH1 *(Figs. 1B and 1C) and MSP suggests that the *CDH1 *promoter is methylated (Figure 2A). Bisulfite sequencing of the intervening CpGs within the MSP product demonstrated that the majority of CpGs in this region of the *CDH1 *promoter are methylated (TMI = 95%, Figure 2B). Sequencing of the same region of the *CDH1 *promoter in low-frequency methylator BT20 cells (which express *CDH1*) revealed that all 19 CpGs are unmethylated (TMI = 0%). Additionally, bisulfite sequencing of the *CDH1 *promoter in hypermethylator MDA-MB-231 cells (which display low level expression of *CDH1 *and exhibit both a methylated and unmethylated *CDH1 *MSP product) revealed one highly methylated allele (TMI = 84%), and one sparsely methylated allele (TMI = 4%, Figure 2B).

The six cell lines of the hypermethylator cluster (BT549, Hs578T, MDA-MB-231, MDA-MB-435S, MDA-MB-436, and MDA-MB-453) were treated with the demethylating agent 5-aza and changes in methylation and expression patterns for five genes (*CEACAM6, CDH1, CST6, ESR1, SCNN1A*) were examined. Representative RT-PCR results are shown in Figure 2C. Whereas these genes are not expressed in the majority of hypermethylator cell lines (Figure 1), treatment with 5-aza results in robust expression (Figure 2C). Bisulfite sequencing of *CDH1, CEACAM6*, and *ESR1 *confirmed that promoter demethylation following 5-aza treatment coincided with gene expression for these genes (data not shown).

### Hypermethylator Phenotype Status is Predicted by Indicator Gene Expression

A Bayesian analysis was performed to evaluate the value of each gene in predicting correctly which of the two clusters a given cell line was sorted. Five genes emerged as excellent individual indicators (predictors) of cluster assignment, having correct assignment values of 75% or greater: *CDH1 *(83%), *CEACAM6 *(CA = 92%), *ESR1 *(75%), *LCN2 *(75%), and *SCNN1A *(92%). These genes individually display excellent sensitivity (range: 71–100%) and specificity (range: 63–86%), good positive predictive value (range: 50–83%), and excellent negative predictive value (range: 67–100%). Additionally, *CST6 *had high sensitivity, specificity, and negative predictive values (75%, 63% and 86%, respectively) and produced 67% correct assignments. Cell lines of the hypermethylator phenotype frequently do not express these genes (hypermethylator cell lines express between 0–2 genes at normal levels). Furthermore, BT549, MDA-MB-453S, and Hs578T cells do not express any of the indicator genes (Figure 1C). In contrast, the cell lines belonging to the low-frequency methylator group frequently express these genes at normal levels (with low-frequency methylator cell lines retaining some level of expression at 3–6 genes, p = 0.00045). MDA-MB-468, MDA-MB-415 and BT20 cells retain detectable levels of expression of 100% (6/6) of these genes (Figure 1C).

### Gene Expression Status Correlates with Promoter Methylation Status Among Breast Cancer Cell Lines

**Figure 3:**
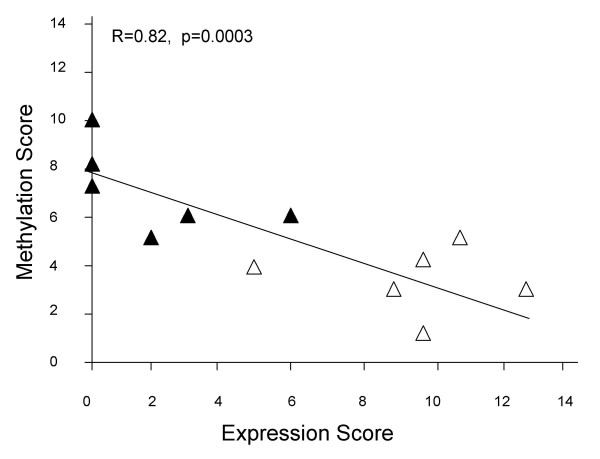
**Gene expression status correlates with promoter methylation status among breast cancer cell lines**. Association between RT-PCR expression and MSP methylation status of the six indicator genes for the 12 breast cancer cell lines. Scores were calculated for *CEACAM6, CDH1, CST6, ESR1, LCN2*, and *SCNNIA*. Hypermethylator cell lines (black triangles) and low-frequency methylator cell lines (white triangles) demonstrate a statistically significant relationship between gene expression status and promoter methylation status.

To examine the relationship between gene expression status and promoter methylation for each of the six indicator genes, an expression score and a methylation score were generated for each cell line. These scores reflect the combined relative expression and the combined relative methylation status for these genes of interest (*CEACAM6, CDH1, CST6, ESR1, LCN2*, and *SCNNIA*). A strong inverse correlation (R = 0.82, p = 0.0003) exists between these two parameters: cell lines with low expression scores tend to have higher methylation scores, and those with high expression scores tend to have low methylation scores (Figure 3). Hypermethylator cell lines exhibit an average expression score of 1.8 ± 1.0, while low-frequency methylator cell lines exhibit an average expression score of 9.7 ± 1.0. This difference in average expression score was significant (p = 0.0003). Likewise hypermethylator cell lines produced an average methylation score that was significantly higher than that for the low-frequency methylator cell lines (7.0 ± 0.7 versus 3.3 ± 0.6, p = 0.003). These results suggest that the loss of gene expression observed in hypermethylator cell lines is a direct consequence of aberrant promoter methylation for the genes of interest.

### DNMT Analysis Reveals Aberrant DNMT Activity and Elevated DNMT Protein Levels among Hypermethylator Cell Lines

**Figure 4:**
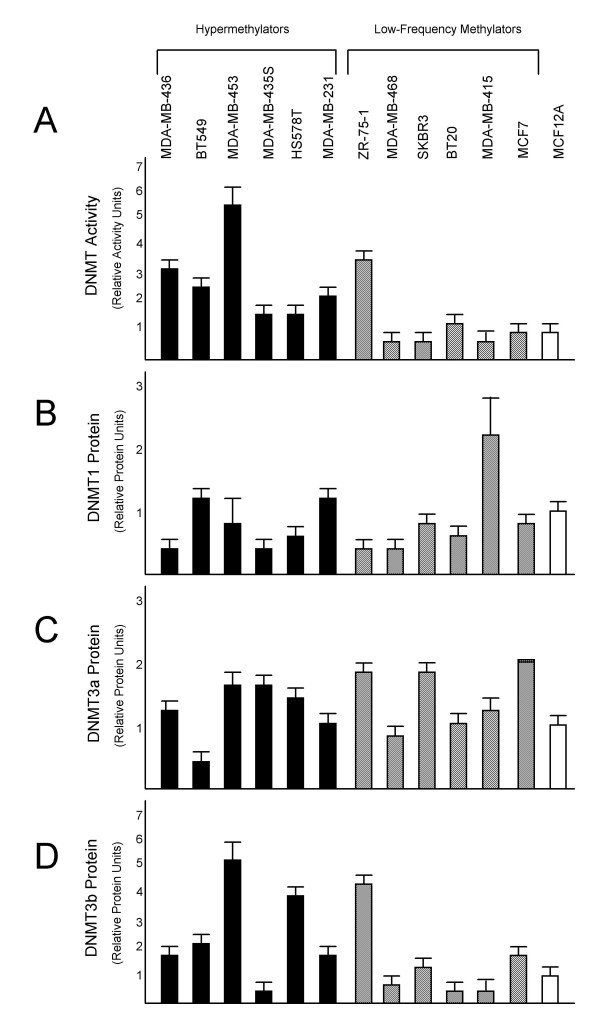
**Analysis of DNA methyltransferase enzymes among putative hypermethylator and low-frequency methylator cell lines**. Results from triplicate determination of total DNMT activity and individual DNMT protein assays are shown. Hypermethylator cell lines are represented by black bars, low-frequency methylators are represented by cross-hatched bars, and MCF12A cells are represented by a white bar. Error bars represent S.E.M. One unit of DNMT activity or DNMT protein level corresponds to the equivalent amount of activity or protein expressed in MCF12A cells. (A) Total DNMT enzymatic activity; (B) DNMT1 protein; (C), DNMT3a protein; and (D), DNMT3b protein.

Hypermethylator cell lines exhibit total DNMT activity levels that are higher than that of low-frequency methylator cell lines and non-neoplastic MCF12A cells (Figure 4A). Each of the hypermethylator cell lines exhibit DNMT activity levels that are ≥ 1.7-fold higher than that of MCF12A cells (Figure 4A), whereas 5/6 (83%) low-frequency methylator cell lines (MDA-MB-468, SKBR3, BT20, MDA-MB-415, and MCF7) exhibit DNMT activity levels that are ≤ 1.4-fold that of MCF12A cells (Figure 4A). The average DNMT activity level for the hypermethylator cell lines (2.9 ± 0.6) is greater than that of the low-frequency methylator cell lines (1.4 ± 0.5), but the difference does not reach significance (p = 0.095, NS). This is due to the level of DNMT activity in ZR-75-1 cells (3.8 ± 0.2), which is much higher than MCF12A cells, making it unlike the other five cell lines in the low-frequency methylator group. When ZR-75-1 cells are excluded, the collective DNMT activity level of the low-frequency methylator group becomes indistinguishable from that of MCF12A cells and significance emerges between the total DNMT activity levels of the hypermethylator and low-frequency methylator groups (p = 0.027).

No significant differences were detected for DNMT1 or DNMT3a protein levels between hypermethylator cell lines, low-frequency methylator cell lines, and MCF12A cells (Fig. 3b–3C). The average DNMT1 protein level for the hypermethylator cell lines (0.8 ± 0.15) and the low-frequency methylator cell lines (0.88 ± 0.29) are indistinguishable from those of MCF12A cells (p = 0.82, NS, Figure 4B). MDA-MB-415 cells overexpress DNMT1 (2.3-fold compared to MCF12A), but the other cell lines exhibit a DNMT1 protein level of 1.3-fold or lower regardless of their methylation status (Figure 4B). Likewise, the average DNMT3a protein level for the hypermethylator cell lines (1.24 ± 0.17) and the low-frequency methylator cell lines (1.39 ± 0.2) are indistinguishable from that of MCF12A cells (p = 0.59, NS, Figure 4C). In contrast to DNMT1 and DNMT3a, the average DNMT3b protein levels for the hypermethylator cell lines are higher (2.5 ± 0.67) than those of the low-frequency methylator cell lines (1.5 ± 0.64, Figure 4D), but this difference was not statistically significant. Among the hypermethylator cell lines, 5/6 (83%) express ≥ 1.7-fold MCF12A levels of DNMT3b protein. In contrast, among the low-frequency methylator cell lines, only ZR-75-1 cells (which also displays high DNMT activity) exhibit an elevated level of DNMT3b protein level expression (Figure 4D). While ZR-75-1 cells display a similar methylation defect to the hypermethylator cells (elevated DNMT3b protein and total DNMT activity), they fail to silence the same methylation-sensitive genes that are methylated in the hypermethylator phenotype cell lines. Thus, ZR-75-1 is more similar to the low-frequency methylator cell lines with respect to gene expression and methylation of the six indicator genes. When the cell line ZR-75-1 is excluded from the low-frequency methylator group, the average DNMT3b protein level for the low-frequency methylator cells is 0.91-fold that of MCF12A cells, approaching significance when compared to the hypermethylator cell lines (p = 0.069).

**Figure 5:**
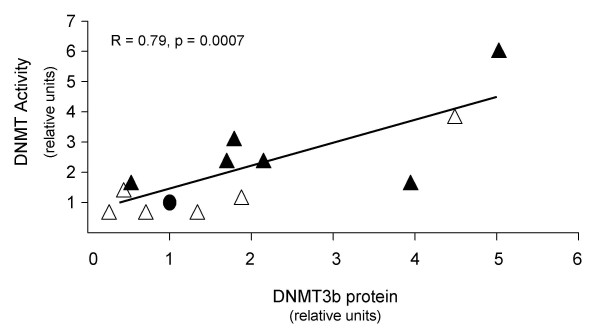
**DNMT activity levels in breast cancer cell lines correlate with DNMT3b expression**. Association between DNMT total activity and DNMT3b protein levels for the 12 breast cancer cell lines and MCF12A cells. Hypermethylator cell lines (black triangles), low-frequency methylator cell lines (white triangles), and MCF12A cells (black circle) demonstrate a statistically significant relationship between DNMT total activity and DNMT3b protein levels.

A correlation analysis was performed to identify significant relationships between DNMT protein levels and DNMT activity among the hypermethylator and low-frequency methylator cell lines. No significant association was found between DNMT activity and DNMT1 or DNMT3a protein levels (R<0.3, NS). However, a strong association (R = 0.79, p = 0.0007) between DNMT activity and DNMT3b protein levels was observed (Figure 5). Statistically significant correlation coefficients were determined for the relationship between DNMT3b protein and DNMT activity for both hypermethylator cell lines (0.71, p = 0.0036), and the low-frequency methylator cell lines (R = 0.90, p = 0.0028). This observation suggests that DNMT3b significantly contributes to total DNMT activity among breast cancer cell lines. Consistent with this suggestion, in cell lines with DNMT activity ≥ 1.8-fold higher than MCF12A cells (n = 7), 86% (6/7) exhibit elevated (≥ 1.7-fold higher than MCF12A) DNMT3b levels. With the exception of ZR-75-1 cells, all of these cell lines belong to the hypermethylator group (MDA-MB-436, BT549, MDA-MB-453, Hs578T, and MDA-MB-231). Significant associations were recognized between DNMT activity and the additive values of (i) DNMT1 and DNMT3b (R = 0.74, p = 0.002), (ii) DNMT3a and DNMT3b (R = 0.74, p = 0.002), and (iii) DNMT1, DNMT3a, and DNMT3b (R = 0.70, p = 0.004). However, these relationships primarily reflect the contribution of DNMT3b to DNMT activity rather than a true additive effect of the various DNMT enzymes. These findings combine to demonstrate significant correlation between hypermethylator status, elevated total DNMT activity, and overexpression of DNMT3b protein.

### Microarray Data Mining Identifies a Distinct Cluster of Basal-like Breast Tumors that Express the Hypermethylation Signature

**Figure 6:**
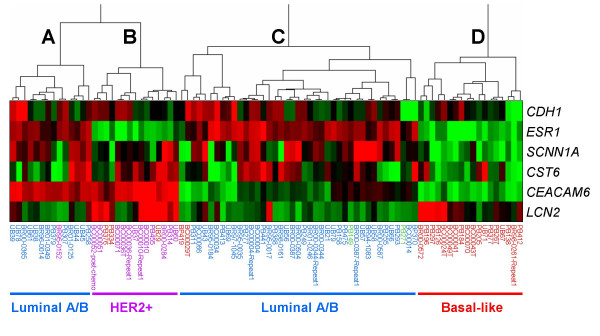
**Basal-like breast cancers express a hypermethylator signature**. Gene expression data from 92 primary human breast tumors from the UNC Microarray Database were subjected to unsupervised cluster analysis based upon the mRNA expression of six genes (*CEACAM6, CDH1, CST6, ESR1, LCN2*, and *SCNN1A*) which define the hypermethylator phenotype. Four tumors failed to cluster and were excluded from further analysis. Gene designations are depicted vertically and tumor designations are shown horizontally. Four clusters (designated A-D) were identified: Cluster A (majority of which are luminal), Cluster B (majority Her2+), Cluster C (majority luminal), and Cluster D (all basal-like). The expression level for each gene is shown relative to the median expression of that gene across all samples, with high expression shown in red and low expression shown in green, while genes with median expression are shown in black. Tumors were classified as luminal A or luminal B (shown in blue), Her2+ (shown in purple), basal-like (shown in red), or normal-like (shown in green).

Gene expression data from the microarray analysis of 92 primary breast tumors (from the UNC Microarray Database) were analyzed for expression of the six genes (*CEACAM6, CDH1, CST6, ESR1, LCN2*, and *SCNN1A*) whose loss characterizes the hypermethylator phenotype among breast cancer cell lines. Unsupervised cluster analysis of these data identified four strong clusters (Figure 6). Eighty-eight of 92 primary breast tumors clustered in this analysis, while four tumors did not cluster and were excluded from further analysis. The 88 breast cancers that clustered in this analysis reflect the following molecular classification: 34/88 (39%) luminal A, 23/88 (26%) basal-like, 16/88 (18%) luminal B, 13/88 (15%) Her2+, and 2/88 (2%) normal-like. Of the four major clusters (designated A-D), Cluster D is composed of 18 tumors that express a hypermethylation signature, characterized by lack of or low expression of the six genes analyzed. Strikingly, 100% (18/18) of these putative hypermethylator tumors are of the basal-like subtype, and this cluster contains 75% (18/24) of basal-like tumors in the dataset. This observation suggests that expression of the hypermethylator phenotype represents a major biological property of basal-like breast cancers. As shown in Figure 6, Clusters A and C (n = 14 and n = 41, respectively) are composed primarily of luminal A and luminal B breast tumors (93% and 90%, respectively), and Cluster B (n = 15) is composed primarily of Her2+ breast tumors (80%).

## Discussion

The CpG island methylator phenotype (CIMP) was first used to describe a distinct subset of colorectal tumors that display high rates of concordant methylation of specific genes [4]. Subsequently, similar epimutational phenomena have been described in a wide range of neoplasms [5-12,14,20]. The results of the present study suggest that a subset of human breast cancer cell lines express a hypermethylator phenotype that is characterized by concurrent methylation-dependent silencing of a number of genes, including a specific set of genes with excellent predictive power (*CDH1, CEACAM6, CST6, ESR1, LCN2*, and *SCNN1A*) that are involved in a wide range of neoplastic processes. *CEACAM6 *is a tumor-related gene that is involved in adhesion, migration, invasion, metastasis, apoptosis, and chemoresistance [21,22], although the implications of its loss in breast cancers is not well understood. Cystatin M (*CST6*) is a recognized breast cancer tumor suppressor gene [23] that was recently reported to be silenced due to promoter hypermethylation in numerous breast cancer cell lines, as well as primary breast tumors [24,25]. E-cadherin (*CDH1*) is a well-known suppressor of invasion/metastasis that functions in the maintenance of cell-cell adhesion [26]. *CDH1 *and *ESR1 *are frequently concurrently methylated in breast tumors [19], a relationship also discernible in the present study. The nuclear hormone receptor *ESR1*, which is silenced by methylation in the majority of estrogen-negative breast tumors [19], may be the foremost important methylation-sensitive gene in breast carcinogenesis, holding important implications for sensitivity to hormone therapy and clinical outcome. Much less well understood is the role of ion transport gene *SCNN1A *in breast carcinogenesis, although its epigenetic regulation in MCF7 cells has previously been noted [24]. *LCN2 *is involved in invasion and metastasis [27], and its expression has been linked to poor prognosis in ER/PR-negative breast tumors [28,29]. Thus, methylation-sensitive genes function in various aspects of the normal biology of the breast epithelium. Therefore, concurrent methylation-dependent silencing of multiple genes in neoplastic breast epithelium (as observed in hypermethylator cell lines) is likely to significantly contribute to tumor biology and behavior.

A previous study that examined methylation patterns of primary breast tumors in search of a hypermethylator phenotype found frequent but essentially equally distributed methylation events at 12 genes among different histologic subsets of neoplasms [15]. These authors concluded that a CpG island methylator phenotype does not occur in breast cancer [15]. The difference in conclusions about the existence of a hypermethylator phenotype in breast cancer between the current study and the earlier report [15] is likely attributable to the number and choice of genes examined in the two studies, as well as the analysis of primary breast tumors versus established cancer cell lines. The previous study did not examine many of the genes that we found to be highly predictive of a hypermethylator phenotype (*CEACAM6, CST6, LCN2*, and *SCNN1A*), but did include several genes (including *GSTP1, RARβ, RB*, and others) which were less useful for predicting the hypermethylator phenotype. Thus, our results are consistent with the previous findings: when the genes are analyzed by Bae et al [15], no distinct hypermethylator phenotype is detectible. It is only through a survey of numerous methylation-sensitive genes that evidence for a hypermethylator phenotype emerges. Additionally, we examined not only genes with conventionally defined CpG islands, but also those with atypical CpG features (such as *CEACAM6*), which have only recently been reported as epigenetically-regulated [24]. Thus, we use the term "hypermethylator phenotype" rather than "CpG island methylator phenotype" to describe the hypermethylation defect in breast cancer cell lines, since the targets of aberrant methylation are not restricted to genes with large CpG islands.

The results of the current study suggest that the mechanism that accounts for the hypermethylator phenotype in human breast cancer cell lines is elevated DNMT activity secondary to overexpression of DNMT3b. DNMT3b protein is significantly elevated in hypermethylator cell lines, and these cells exhibit aberrantly increased DNMT activity and correspondingly high rates of methylation-dependent gene silencing compared to both low-frequency methylator cells and non-neoplastic counterparts. These results are in agreement with those of other recent studies, in which aberrant DNMT3b overexpression was implicated in the methylation abnormalities of breast cancers [30] and other cancers [31]. Tumor cells exhibiting DNMT3b overexpression are likely to exhibit methylation-based aberrant gene expression; one study showed that breast tumors that overexpress DNMT3b are more likely to be *ESR1*-negative, display increased proliferation, and be associated with poor patient prognosis [30]. Thus, it seems reasonable to expect that aberrant expression of DNMT3b protein may produce significant differences in tumor biology for breast tumors of the hypermethylator phenotype. In addition to the six hypermethylator cell lines which had elevated DNMT3b protein and total DNMT activity, one low-frequency methylator cell line (ZR-75-1) exhibited a similar hypermethylation defect. However ZR-75-1 cells retain expression of a number of epigenetically-regulated genes, making it functionally similar to other low-frequency methylator cell lines. A number of explanations may account for this apparent discrepancy: ZR-75-1 cells may methylate other epigenetically-regulated genes which were not surveyed in the present study; alternatively ZR-75-1 cells may possess the same functional defect in the DNMT machinery as cells of the hypermethylator phenotype but express additional repressor proteins which block the methylation capacity of the overabundant DNMT3b protein. Additional studies will be required to resolve these possibilities. The detection of a hypermethylator phenotype in breast cancer cell lines constitutes a first step towards determining if a hypermethylation defect can be identified in primary breast neoplasms in vivo. If a subset of primary breast cancers express a hypermethylator phenotype, we would predict these tumors to differentially express other important characteristics related to tumor biology/behavior and patient outcome. This is the case in colorectal cancer, where CIMP status is associated with various clinical features [32-34]. Likewise, CIMP-positive neuroblastomas, esophageal tumors, and leukemias tend to have poorer prognosis and are associated with significantly higher relapse and mortality rates [12,35,36].

Our findings suggest that breast cancer cell lines that express the hypermethylation defect correspond to estrogen-receptor negative tumors, suggesting that the hypermethylator phenotype cosegregates with a subset of breast cancers (ER-negative) that tend to have poor prognosis [37]. A number of molecular subtypes of breast cancer have been described (including luminal A, luminal B, HER2+ and basal-like), and these different subtypes correlate with important differences in tumor biology, clinical behavior, and patient survival. Luminal A and luminal B tumors are ER-positive and respond better to treatment, resulting in better long-term patient outcome compared to the ER-negative basal-like and HER2+ subtypes [38]. Our microarray data mining analysis of primary breast cancer gene expression suggests that the hypermethylation defect observed in breast cancer cell lines can also be identified in primary tumors. Preliminary investigation of a limited dataset (n = 88 tumors) identified a strong cluster of tumors that express the hypermethylator signature (Figure 6), with low levels of expression of the six genes of interest (*CDH1, CEACAM6, CST6, ESR1, LCN2*, and *SCNN1A*). All of the tumors in this cluster were classified as basal-like, and 75% of the basal-like tumors in the dataset expressed the hypermethylation signature. This observation suggests that the hypermethylator defect represents a biological property of basal-like breast cancers. Basal-like breast tumors make up ~25% of all breast cancers but contribute disproportionately to breast cancer deaths as they tend to display more aggressive tumor characteristics such as increased size, rapid tumor growth, increased rate of metastasis, higher incidence of relapse, and lower overall patient survival [39,40]. In has also been observed that this subtype of breast cancer is overrepresented in young, African-American women [41]. These tumors lack expression of the hormone growth factor receptor genes (ER and PR) that are targeted by some drug regimens, eliminating options for targeted therapy. While further studies are needed to understand fully the relationship between basal-like breast cancers and the hypermethylator phenotype, recognition of this fundamental biological property of the basal-like breast cancers may present new molecular targets for development of novel treatment strategies.

## Conclusion

Unraveling the complexities of this hypermethylation defect in neoplastic breast disease holds important implications for cancer diagnosis, identification of new targets for therapy, and development of new strategies for clinical management. Since overexpression of DNMT is thought to be an early event in carcinogenesis [42-44], elevated DNMT3b protein (which characterizes the hypermethylator phenotype in vitro) may constitute an important biomarker for early detection in patients developing breast tumors of the hypermethylator phenotype. Furthermore, the various proteins and enzymes of the DNA methylation machinery may represent novel targets for breast cancer therapy. It follows that patients with breast cancer of the hypermethylator phenotype may benefit significantly from a targeted demethylation treatment as an adjunct to standard chemotherapeutic regimens. Epigenetic chemosensitization has been shown to improve the efficacy of standard chemotherapeutics against tumor cells with known methylation defects [45,46], and evidence suggests that chemotherapeutic resistance can be overcome with demethylating treatment in certain cases [47]. While more research needs to be done to fully understand the clinicopathological implications of the hypermethylator phenotype in primary breast tumors, the existence of a subset of breast cancer cells with aberrant DNA methylation and other epimutations that are potentially reversible holds promise for better diagnosis and improved treatment.

## Methods

### Cell Culture, RNA, and DNA Preparation

Human breast cancer cell lines BT20 (ATCC# HTB19), BT549 (HTB122), Hs578T (HTB126), MCF7 (HTB22), MDA-MB-231 (HTB26), MDA-MB-415 (HTB128), MDA-MB-435S (HTB129), MDA-MB-436 (HTB130), MDA-MB-453 (HTB131), MDA-MB-468 (HTB132), SKBR3 (HTB30), and ZR-75-1 (CRL-1500) were obtained from the Tissue Culture Core Facility of the University of North Carolina Lineberger Comprehensive Cancer Center (Chapel Hill, NC), and the normal breast epithelial cell line MCF12A [48] (CRL-10782) was obtained from the American Type Culture Collection [49]. Cell lines were propagated in growth medium specified by ATCC. Growth medium was refreshed three times weekly, and cell cultures were harvested for RNA preparation at confluency using the method of Chomczynski and Sacchi [50], modified to utilize TRIzol Reagent (Invitrogen Life Technologies, Carlsbad, CA), according to the manufacturer's protocol. Cell lines selected for treatment with the demethylating agent 5-aza-2'-deoxycytidine (Sigma Chemical Company, St. Louis, MO) were propagated in the appropriate ATCC-recommended growth medium containing 250 nM 5-aza (with refreshing three times weekly) for a total of three weeks, before RNA isolation. As described previously [24], the concentration of 5-aza used in this study is 4–6-fold lower than traditional methods which allows for long term 5-aza exposure without the typically encountered cytotoxic effects [51,52]. Isolated RNA was stored at -20°C as an ethanol precipitate prior to use for RT-PCR. Genomic DNA from 2 × 10^6 ^cultured cells was isolated using the Puregene DNA Purification Kit (Gentra Systems, Minneapolis, PA). Bisulfite modification of genomic DNA was performed using a procedure adapted from Grunau et al [53], as described previously [24].

### Semi-quantitative RT-PCR

Sixty-four genes were selected for analysis in this study based on their status as marker genes for CIMP in other tumor systems or genes that are known to be methylated in breast cancer specifically (Table 1). Total RNA (2 μg) collected from each cell line was reverse-transcribed into cDNA using Superscript II Reverse Transcriptase (Invitrogen Life Technologies, Carlsbad, CA) and oligo(dT) as the primer, according to standard methodology. Gene-specific oligonucleotide primers were designed using Primer3 software [54] and were synthesized by the UNC Oligodeoxynucleotide Synthesis Core Facility (Chapel Hill, NC) based upon the known cDNA sequences [55] for selected mRNAs of interest. The RT-PCR primer sequences and thermocycling conditions for *CEACAM6, CST6, LCN2*, and *SCNN1A *have been described previously [24], while those for *CDH1 *and *ESR1 *are as follows: *CDH1*, forward 5'-TCT-TGC-TGT-TTC-TTC-GGA-GG and reverse TGA-CTC-TGA-GGA-GTT-CAG-GG (60°C, 30 cycles, 380 bp product); *ESR1*, forward 5'-TTG-TCC-CAT-GAG-CAG-GTG-CC and reverse 5'-GTA-TGC-ATC-GGC-AAA-AGG-GC (58°C, 30 cycles, 201 bp product). Verification of equal cDNA template concentrations between samples was accomplished using *β-actin *primers (forward 5'-AGA-GAT-GGC-CAC-GGC-TGC-TT and reverse 5'-ATT-TGC-GGT-GGA-CGA-TGG-AG,). PCR reactions were performed in a 50 μl total volume of buffer containing 50 mM KCl, 10 mM Tris-HCl (pH 8.3), 1.5 mM MgCl_2_, 0.001% gelatin, 200 μM of each dNTP (EasyStart Micro 50 PCR-mix-in-a-tube, Molecular BioProducts, San Diego, CA), 0.4 μM of each primer, and 2.5 units AmpliTaq enzyme (Perkin Elmer/Cetus, Foster City, CA). Reactions were carried out in an Eppendorf Mastercycler Thermocycler as follows: 30–35 cycles at 94°C for denaturing (1 minute), 58–65°C for annealing (1.5 minutes), and 72°C for extension (2 minutes). PCR products were fractionated on 2% agarose gels containing 40 mM Tris-acetate/1.0 mM EDTA and visualized by ethidium bromide staining.

### Quantitative Real-time PCR

Total RNA samples (2 μg) from cell lines of interest were DNAase treated (Promega, Madison, WI), purified using the Qiagen Rneasy mini-kit (Qiagen, Valencia, CA), and reversed transcribed using the High Capacity cDNA Archive Kit (Applied Biosystems, Foster City, CA) according to the manufacturer's protocol. Real-time primers and probes for *CDH1 *(Assay ID: Hs00170423_m1), *CEACAM6 *(Hs00366002_m1), *CST6 *(Hs00154599_m1), *ESR1 *(Hs00174860_m1), *LCN2 *(Hs00194353_m1), *SCNN1A *(Hs00168906_m1), and *β-actin *(Hs99999903_m1) were purchased from Applied Biosystems (Foster City, CA). Reactions were carried out using TaqMan Universal PCR Master Mix (Applied Biosystems, Foster City, CA) and the following amplification conditions: 95°C for 10 min, 40 cycles of 95°C for 15 sec, and 60°C for 1 min. Gene expression levels were normalized using *β-actin *for each cell line and differences in gene expression were determined using the comparative Ct method described in the ABI Prism 7700 User Bulletin #2 (Applied Biosystems, Foster City, CA).

### Cluster Analysis of Breast Cancer Cell Lines Based Upon Gene Expression Patterns

Expression levels for genes of interest were analyzed by RT-PCR using cDNA templates derived from 12 breast cancer cell lines and normal MCF12A breast epithelial cells. RT-PCR results for breast cancer cell lines were expressed on a discrete scale (none, low, medium, high) relative to the expression levels of MCF12A cells. Genes from the original panel of 64 that were not expressed in MCF12A cells (n = 16) were omitted from the cluster analysis, to ensure that cancer-specific methylation events were captured. The expression data were mapped to a quantitative scale (0, 1, 2, 3) for clustering purposes. For some analyses, a combined expression score was generated for each cell line by adding the quantitative RT-PCR expression levels of genes of interest. Clustering of cell lines was carried out with SAS/STAT PROC CLUSTER (SAS Institute, Cary, NC) using complete linkage with 5% trimming and no squaring of distance. Kernel density estimation for trimming used the 5 nearest neighbors.

### Methylation-specific PCR, Cloning, and Sequencing

MSP reactions were carried out in EasyStart Micro 50 PCR-mix-in-a-tube (Molecular BioProducts, San Diego, CA) using bisulfite converted DNA template (described above). The primers and thermocycling conditions for *CDH1, CST6*, and *ESR1 *genes have been described previously [25,56,57]. MSP primers directed against methylated and unmethylated alleles of *CEACAM6, LCN2*, and *SCNN1A *are as follows: methylated *CEACAM6*, forward primer 5'-AGG-GCG-GGT-CGT-TTT-GTT-AT, reverse primer 5'-TCA-CGT-AAA-TCA-TAA-ATA-CGA-TCT-CT (58°C, 35 cycles, 174 bp product); unmethylated *CEACAM6*, forward primer 5'-AGG-GTG-GGT-TGT-TTT-GTT-AT, reverse primer 5'-TCA-CAT-AAA-TCA-TAA-ATA-CAA-TCT-CT (55°C, 35 cycles, 174 bp product); methylated *LCN2*, 5'-CGA-GAG-TTA-TTG-CGT-TTA-GTC-GA, reverse primer 5'-CGA-ATA-AAT-CAC-GAA-ATC-AAA-AAT-TCG-A (60°C, 35 cycles, 273 bp product); unmethylated *LCN2*, forward primer 5'-AGA-GTT-ATT-GTG-TTT-AGT-TGA-GGA, reverse primer 5'-CAA-ATA-AAT-CAC-AAA-ATC-AAA-AAT-TCA-A (55°C, 35 cycles, 273 bp product); methylated *SCNN1A*, forward primer 5'-TCG-GGA-GTT-TTT-TTT-TTT-TCG-GA, reverse primer 5'-CCG-CCC-GCT-AAC-CGA (56°C, 40 cycles, 135 bp product); unmethylated *SCNN1A*, forward primer 5'-TTG-GGA-GTT-TTT-TTT-TTT-TTG-GA, reverse primer 5'-AAC-CCA-CCC-ACT-AAC-CAA (56°C, 40 cycles, 135 bp product). PCR products were fractionated on 2% agarose gels and visualized by ethidium bromide staining. For some analyses, MSP results were converted from a discrete scale (unmethylated product only, both methylated and unmethylated products, or methylated product only) to a quantitative scale (0, 1, 2) in order to generate a methylation score for each cell line that reflects the combined methylation status of select genes of interest.

Bisulfite-converted DNA was amplified using MSP primers directed to specific segments within the promoter regions and/or exon 1 of selected genes. A portion of each PCR product (1 to 5 μl) was cloned into pGEM-T Easy Vector (Promega, Madison, WI). Colonies (n = 5–10) were selected per gene segment and expanded in liquid culture. Plasmid DNA was purified using the Wizard Plus Miniprep DNA Purification Kit (Promega, Madison, WI), prior to digestion with NcoI and NdeI (New England Biolabs, Beverly, MA) to confirm the presence of the cloned insert. Validated clones were sequenced using the universal M13R3 primer with an Applied Biosystems automated sequencer at the UNC Genome Analysis Facility (Chapel Hill, NC). In some cases, the sequencing results are expressed as total methylation index (TMI), which is calculated by dividing the number of methylated CpGs observed by the total CpGs analyzed for a given gene segment of interest [58].

### DNA Methyltransferase Analysis of Human Breast Cancer Cell Lines

Total DNA methyltransferase activity was measured using EpiQuik DNA Methyltransferase Activity/Inhibition Assay Kit (Epigentek, Brooklyn, NY) as previously described [59], using nuclear extracts from 12 human breast cancer cell lines and MCF12A cells. Nuclear extracts were isolated using the EpiQuik Nuclear Extraction Kit (Epigentek, Brooklyn, NY) and 3 μl of nuclear extract was added to each reaction well, according to manufacturer's protocol. The final volume of nuclear extract yield was used to normalize the assay results for differences in cell number. Nuclear extracts were incubated with methylation substrate for 1 hour at 37°C, and then exposed to the capture antibody for 60 minutes and the detection antibody for 30 minutes, at room temperature. Absorbance was determined using a microplate spectrophotometer at 450 nm, and DNMT activity (O.D./h/ml) was calculated according to the following formula: (Sample OD – blank OD)/(sample volume × 1000), according to manufacturer's instructions. Results are given in activity units expressed relative to the activity level detected in MCF12A cells.

Nuclear extracts were assayed for individual DNMT proteins of interest (DNMT1, DNMT3a, or DNMT3b) using the Epiquik DNMT1, -3a, and -3b assay kits, respectively (Epigentek, Brooklyn, NY). Protein standards of known concentration (30 ng, 20 ng, 10 ng, and 2 ng) were included to generate a standard curve. The amount of DNMT protein was calculated as follows: DNMT protein (ng/ml) = (Sample OD – blank OD/standard slope) × sample dilution, according to the manufacturer's instructions, and are expressed relative to the protein levels of MCF12A cells.

### Cluster Analysis of Gene Expression

The publicly available microarray dataset utilized in this study is available online at the UNC Microarray Database [60] and includes gene expression data for 92 primary breast tumors analyzed in previous studies [61-64]. Clustering of transcripts was carried out with SAS (PROC CLUSTER) based on distance of the log ratio values using complete linkage with 5% trimming. The kernel density estimation for trimming used the 10 nearest neighbors.

### Statistical Analysis

The values for the mean and S.E.M. were calculated using the statistical function of KaleidaGraph Version 3.5 (Synergy Software, Essex Junction, VT). Statistical significance was determined using an unpaired t-test (KaleidaGraph). Error bars depicted represent S.E.M. P values for correlation coefficients (R values) were calculated using VasserStats Significance of Correlation Coefficient Calculator [65]. The Bayesian analysis was performed as described previously [66] and the percentage of correct assignments, as well as sensitivity, specificity, and positive and negative predictive values were calculated.

## Authors' contributions

JDR carried out the majority of expression, methylation, and DNMT experiments and analyses, and drafted the manuscript. AGR performed select DNA and RNA isolations from the breast cancer cell lines and performed methylation analyses of *CST6*. WDJ performed the unsupervised cluster analysis on RT-PCR expression data and provided support for additional statistical analyses. WBC conceived of and designed the study, participated in its experimental design and interpretation of results, and helped edit the manuscript. All authors read and approved the final manuscript.
